# Association of Dyslipidemia with Renal Cell Carcinoma: A 1∶2 Matched Case-Control Study

**DOI:** 10.1371/journal.pone.0059796

**Published:** 2013-03-25

**Authors:** Chunfang Zhang, Luping Yu, Tao Xu, Yichang Hao, Xiaowei Zhang, Zhenhua Liu, Yunbei Xiao, Xiaofeng Wang, Qiang Zeng

**Affiliations:** 1 Department of Clinical Epidemiology, Peking University People’s Hospital, Beijing, China; 2 Department of Urology, Peking University People’s Hospital, Beijing, China; 3 International Medical Center, Chinese PLA General Hospital, Beijing, China; Children’s National Medical Center, United States of America

## Abstract

Abnormal serum lipid profiles are associated with the risk of some cancers, but the direction and magnitude of the association with renal cell carcinoma is unclear. We explore the relationship between serum lipids and renal cell carcinoma via a matched case-control study. A 1∶2-matched case-control study design was applied, where one renal cell carcinoma patient was matched to two non-renal-cell-carcinoma residents with respect to age (±0 year) and gender. Cases (n = 248) were inpatients with a primary diagnosis of renal cell carcinoma, confirmed by pathology after operations. Controls were sampled from a community survey database matched on age and gender with cases, 2 controls for each case. Stratified Cox proportional hazard regression analysis was used to obtain hazard ratios and corresponding 95% confidence intervals of lipids level and dyslipidemia for the risk of renal cell carcinoma. Elevated serum cholesterol (p<0.001), LDL cholesterol (p<0.001), and HDL cholesterol (p = 0.003) are associated with decreased hazard of renal cell carcinoma, adjusting for obesity, smoke, hypertension and diabetes. However, risk caused by hTG showed no statistical significance (p = 0.263). This study indicates that abnormal lipid profile influences the risk of renal cell carcinoma.

## Introduction

Kidney cancer is a relatively rare disease in human, accounting for about 2% of all cancers worldwide. Approximately 150 000 new cases and 78 000 deaths from the disease occur annually [1]. In the USA, more than 80% of kidney cancers are renal cell carcinomas arising from the renal parenchyma. Increasing incidence and mortality rates for kidney cancers, particularly renal cell carcinomas, have been reported in some countries over the past few decades [2], [3], [4], [5], [6]. China has had an obviously rising trend in kidney cancer incidence since the 1980s, with a rate of 4.7% annually for men and 3.6% for women [7].

Several well-established life-style risk factors, such as BMI, hypertension, and smoking, have been identified as potentially predisposing to renal cell carcinoma development [8]. A history of diabetes mellitus is reported to link with renal cell carcinoma in several cohort studies, although a role independent from that of obesity or hypertension has not been demonstrated conclusively [9], [10], [11], [12]. Subjects with these conditions also have abnormal serum lipid metabolism. Results from a number of case-control studies have indicated that the commonly prescribed cholesterol-lowering drugs, the statins, may reduce the risk of certain cancers. A study conducted in US veterans found that patients who were being treated with statins for control of dyslipidemia appeared to have a significantly reduced risk of kidney cancer, while serum triglyceride concentrations showed positive association with renal cancer in men [13]. Alsheikh-Ali et al. reported an inverse association between on-treatment LDL-C levels and cancer incidence in statin-treated patients enrolled in large randomized controlled trials [14], [15].

However, relationships between serum lipid concentration or dyslipidemia and renal cell carcinoma have not been established yet. Therefore, we conducted this 1∶2 matched case-control study to determine whether a direct association exists between dyslipidemia and renal cell carcinoma, after adjusting for well-recognized life-style risk factors such as obesity, smoking, hypertension, and diabetes.

## Materials and Methods

### Ethics Statement

Ethical approval was obtained from the Clinical Research Ethics Committee of Peking University People’s Hospital. The Declaration of Helsinki was adhered to and written informed consent was obtained from all the subjects for data analysis and research purposes.

### Study Design

In this study, we used a 1∶2-matched case-control study design, where one renal cell carcinoma patient was matched to two non-renal-cell-carcinoma residents on age (±0 year) and gender. Medical record data from April, 2007 to December, 2010 for patients admitted to Peking University People’s Hospital, an affiliated hospital of Peking University, were utilized in the study. Cases (n = 248) were defined as inpatients with a primary diagnosis of renal cell carcinoma that was confirmed by pathology after operations. Data at the time of admission for demographics (ethnicity, age, gender, BMI), smoking status, history of hypertension and diabetes, blood pressure, fasting glucose, statin taking and concentrations of plasma lipids (total cholesterol (TC), triglyceride (TG), HDL cholesterol (HDL-C), and LDL cholesterol (LDL-C)) were obtained from medical records. Controls were sampled from a 2007 community survey database matched for age and gender with the study cases, using two controls for each case. All controls (n = 496) were defined as community residents without diagnosis of renal cell carcinoma at the time of or prior to the study. All relevant data as cases were then extracted from the database.

### Biochemical Tests

Fasting blood samples were taken for measurement of plasma glucose and lipids profile (TC, TG, HDL-C, and calculated LDL-C). Plasma lipids were measured by enzymatic methods on a Hitachi 7600 automatic clinical chemistry analyzer (Boehringer Mannheim, Mannheim, Germany) using reagent kits supplied by the analyzer manufacturer. LDL cholesterol was calculated using the Friedewald equation [16]. The performance precision of these assays was within the manufacturer’s specifications. All measurements were performed by skilled clinical laboratory examiners at our hospital.

### Data Manipulating and Statistical Analysis

Dyslipidemia was defined as follows: 1) hypercholesterolemia (hTC): TC> = 5.18 mmol/L (200 mg/dL); 2) hypertriglyceridemia (hTG): TG> = 1.70 mmol/L (150 mg/dL); 3) high LDL-cholesterolemia (hLDL-C): LDL-C> = 3.37 mmol/L (130 mg/dL); 4) Low HDL-cholesterolemia (lHDL-C): HDL-C<1.04 mmol/L (40 mg/dL); 5) Dyslipidemia: any of hypercholesterolemia or hypertriglyceridemia or low HDL-cholesterol. Diabetes (diabetic or being treated for diabetes/not diabetic), obesity (obese (BMI≥24)/not obese (BMI<24)), smoking (former or current smoker/non-smoker), and hypertension (hypertensive or being treated for hypertension/not hypertensive) were incorporated in the model as dichotomous covariates.

Statistical analysis was performed using R software (version 2.11.0). Matching on age and gender was performed using R programming. After the matched dataset was generated, stratified Cox proportional hazard regression analysis was performed on the matched pairs to obtain hazard ratios (HRs) and corresponding 95% confidence intervals (CIs) of lipid levels and dyslipidemia for the risk of renal cell carcinoma. All statistical tests were two-sided, and significance level was set as 0.05. Data are presented as mean±SD or N (percentage), as appropriate. Lipids level was analyzed as continuous variables with other covariates, while dyslipidemia status was used as a dichotomous variable. Stratified Cox proportional hazard regression analysis was performed using natural logarithm transformed TG values (lnTG) because the original TG values were substantially skewed. We estimated the HRs of plasma lipid levels and dyslipidemia for the risk of renal cell carcinoma by introducing TC, hTC, lnTG, hTG, LDL-C, hLDL-C, HDL-C, and lHDL-C into the Cox regression models one at a time, while adjusting for obesity, smoking, hypertension, and diabetes.

## Results

### Characteristics of the Cases and Controls

In total, 744 Chinese Han ethnic subjects were enrolled in our study: the case/control ratio was 248/496 and the male/female ratio was 332/166; subjects had an average age of 58±13 years (range from 15 to 88). The clinical and biochemical characteristics of the study subjects are shown in Table 1. Compared with the controls, the renal cell carcinoma cases had higher triglyceride level, lower total cholesterol, LDL cholesterol and HDL cholesterol levels, and were more likely to smoke and to be hypertensive and diabetic. Renal cell carcinoma cases were mostly (226/248, 90.96%) the conventional cell type. Details of the carcinomas are described in Table 2.

**Table 1:** Description and comparison of clinical and biochemical characteristics of the study subjects*.

	Controls	Cases	P-value†
N	496	248	
Age	57±13	57±13	
Gender (male)	332 (66.94)	166 (66.94)	
Smoking	317 (63.91)	104 (41.94)	<.0001
Height(cm)	164.24±8.49	167.64±7.33	<.0001
Weight(kg)	68.38±11.99	71.30±11.92	0.0020
BMI (kg/m2)	25.29±3.61	25.31±3.57	0.9274
Obesity	98 (19.76)	53 (21.37)	0.6072
Hypertension	246 (49.60)	133 (53.63)	0.2962
Diabetes	60 (12.10)	64 (25.81)	<.0001
Statin-taking	99 (20.00)	40 (16.10)	0.2063
TC (mmol/L)	4.91±0.90	4.53±1.07	<.0001
hTC	191 (38.51)	62 (25.00)	.0003
TG (mmol/L)	1.63±1.51	1.67±1.15	.0808
hTG	144 (29.03)	92 (37.10)	.0255
LDL-C (mmol/L)	2.88±0.87	2.65±0.85	<.0001
hLDL-C	130(26.21)	47(18.95)	<.0001
HDL-C (mmol/L)	1.29±0.34	1.13±0.32	<.0001
lHDL-C	211 (42.54)	136 (54.84)	.0017

* Quantitative data were presented as mean±SD, and categorical data as N (percentage);

† P-values were obtained from univariate Cox regression.

**Table 2:** Description of the renal cell carcinoma.

	N (%)
Histology subtypes	
Conventional	226 (90.96)
Papillary	10 (4.22)
Chromophobe	6 (2.41)
Unclassified	6 (2.41)
Size	
< = 4.0	164 (66.04)
∼7.0	61 (24.53)
>7.0	23 (9.43)
Side	
Left-sided	139 (56.02)
Right-sided	108 (43.37)
Two-sided	1 (0.60)
Grading	
Grade I	26 (10.38)
Grade I–II	28 (11.32)
Grade II	126 (50.94)
Grade II–III	35 (14.15)
Grade III	26 (10.38)
Grade III–IV	5 (1.89)
Grade IV	2 (0.94)
TNM staging	
T	
1	96 (38.55)
1a	63 (25.30)
1b	37 (15.06)
2	18 (7.23)
3	10 (4.22)
3a	12 (4.82)
3b	7 (3.01)
x	4 (1.81)
N	
0	239 (96.39)
1	9 (3.61)
M	
0	238 (95.78)
1	10 (4.22)

### Plasma Lipid Levels and Dyslipidemia for the Risk of Renal Cell Carcinoma

**Figure 1:**
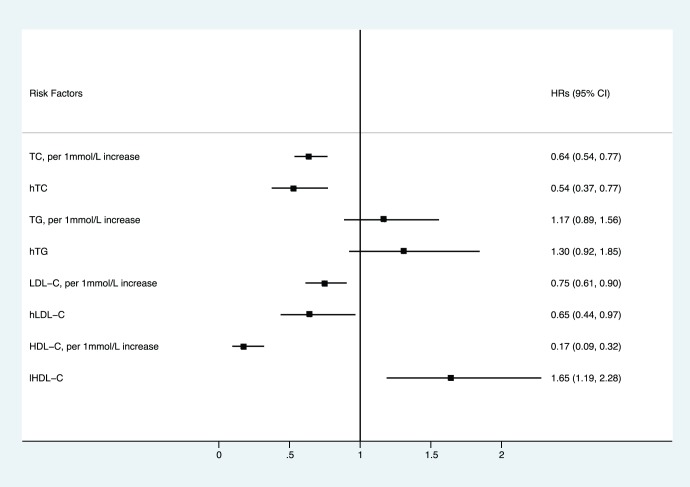
Hazard ratios of plasma lipids and dyslipidemia from stratified Cox regression models. Multifactor adjustment was for obesity, smoke, hypertension, and diabetes. Black squares represent the hazard ratios, and error bars indicate the 95% confidence intervals (CIs).

As shown in Fig. 1, when plasma lipid level and dyslipidemia status are introduced into models one at a time, while adjusting for obesity, smoking, hypertension, and diabetes, the risk of renal cell carcinoma decreases with elevation of TC, LDL-C and HDL-C levels (HR (95% CI) of TC = 0.642 (0.535, 0.769), of LDL-C = 0.745 (0.613, 0.905), of HDL-C = 0.175 (0.095, 0.320) ). The risk caused by elevated TG levels shows no statistical significance (HR (95% CI) = 1.175 (0.886, 1.558)).

A decreased risk of renal cell carcinoma appeared with hTC (HR (95% CI) = 0.537 (0.374, 0.772)) and hLDL-C (HR (95% CI) = 0.649(0.442,0.971)) and an increased risk with lHDL-C (HR (95% CI) = 1.646 (1.187, 2.282)). Risk caused by hTG showed no statistical significance (HR (95% CI) = 1.305 (0.922, 1.846)).

### Sensitivity Analysis

We evaluated the impact of control selection on the estimates by re-analyzing our data using alternate control groups. We observed no considerable variation in the association between plasma lipid concentrations and dyslipidemia with renal cell carcinoma.

## Discussion

The results of our 1∶2 matched case-control analyses indicate that serum lipid profile is associated with renal cell carcinoma. Subjects with lHDL-C have greater odds of renal cell carcinoma than do subjects without, whereas subjects with hTC/hLDL-Chave lower odds.

The impact of high serum cholesterol on cancer incidence is inconsistent. Several studies have reported that cancer incidence [17], [18] and cancer mortality [19], [20] were lower in men with high baseline levels of TC. While this inverse association was seen in the majority of previous studies, others found cancer risk showed no relation at all [21], or a U-shaped association, with TC levels [22], [23]. Our study suggests that a population with elevated blood cholesterol concentration or hypercholesterolemia, has a lower renal cell carcinoma hazard.

In the wake of the extensive use of lipid-lowering statin drugs in cardiovascular diseases, the relationship between low density lipoprotein cholesterol levels and cancer increasingly draws more attention, although it remains controversial. One study has shown an inverse and significant association between on-treatment LDL cholesterol levels and cancer in patients treated with statins, and this association remained even after adjusting for age and other variables [15]. However, a similar relationship was noted between LDL cholesterol levels and incident cancer among control patients not treated with statins; statin therapy, despite significantly reducing LDL-cholesterol levels, was not associated with an increased risk of cancer. In contrast, a Mendelian randomization study in Denmark found that low plasma levels of LDL cholesterol were robustly associated with an increased risk of cancer (kidney cancers included) [24]. However, genetically reduced LDL cholesterol levels (due to polymorphisms that were associated with lifelong reduced plasma LDL cholesterol levels) were not associated with increased risk, which suggested that low LDL cholesterol levels per se do not cause cancer [24]. Only limited studies have examined the relationship between LDL cholesterol levels and the risk of renal cell cancer. Our findings suggest that the renal cell cancer hazard decreasedby 25.5% as the LDL-C concentration increases by 1 mmol/L. The population with a high LDL-cholesterolemia has a lowerhazard for renal cell cancer than does the normal LDL-cholesterolemia population, after adjusting for obesity, smoking, hypertension, and diabetes. Thus, an abnormally high LDL-C level may indicate a lowet risk of renal cell carcinoma.

Studies have suggested an inverse association between high HDL-cholesterol levels and the epidemiology of breast, lung cancer, non-Hodgkin’s lymphoma, and overall cancer risk [25], [26], [27], [28], [29]. Our study suggests a greater hazard of renal cell carcinoma of 165% among low HDL-cholesterolemia patients, adjusting for obesity, smoking, hypertension, and diabetes. Biological mechanisms that might link low serum levels of HDL-cholesterol with cancer are not well established [28]. The function of HDL-cholesterol in reverse cholesterol transport is important in development of atherosclerosis; however, it is not obvious how this function of HDL-cholesterol could influence carcinogenesis [30]. HDL regulation of cell cycle entry through a mitogen activated protein kinase-dependent pathway [31] and apoptosis [32], modulation of cytokine production, and an anti-oxidative function [33] has been considered and is biologically plausible. However, it is also plausible that this association reflects the effect of factors that are associated with both HDL-cholesterol and risk of cancer, such as inflammation, which is known to reduce HDL-cholesterol and likely increases risk of renal cell carcinoma.

Our study had some unavoidable limitations. First, renal cell carcinoma might cause dyslipidemia (decreased serum cholesterol, decreased HDL-C, and elevated LDL-C), with potential mechanisms including effects on cholesterol absorption, transport, metabolism, or utilization. Previous studies have supported the notion that cancers affect cholesterol metabolism or utilization by showing that plasma cholesterol levels are inversely associated with tumor mass of hematological cancers and that plasma cholesterol levels revert to normal after cancer remission [34], [35]. Consideration of confounding factors, such as variety of food intake and other serum contents is also important as these could both cause dyslipidemia and increase the risk of cancer. Other potential limitations of this study include selection bias and misclassification of serum lipids levels. The study cases were selected using medical records data to include inpatients with a primary diagnosis of malignant renal cell carcinoma, which was confirmed by pathology after operations, while controls were randomly sampled from a community-based survey dataset; this should largely exclude any important selection bias. Some participants had a relatively normal serum lipid profile because they were taking statin to lower cholesterol and thus were classified into the non-dyslipidemia group, which would tend to make the estimation of the association between dyslipidemia and risk of cancer more conservative. However, even after we excluded all statin-treated participants from the analysis, elevated serum cholesterol, HDL-C and LDL-C remained associated with an decreased risk of cancer. The final limitation of this study was that all participants were Chinese; therefore, our results may not necessarily apply to other races or ethnicities.

As a metabolic disease [36], renal cell carcinoma may have links to obesity, smoking, hypertension, diabetes, and abnormal serum lipid metabolism. Our study indicates that elevated serum cholesterol, HDL-C and LDL-C may be associated with an decreased risk of cancer, after adjusting for obesity, smoking, hypertension, and diabetes. Further studies on the potential role of serum lipids as an etiologic factor of renal cell carcinoma will be of substantial importance to public health, considering the high prevalence of dyslipidemia in developed and developing countries.
